# Supplementary material for: Kinematic Parameters That Can Discriminate in Levels of Functionality in the Six-Minute Walk Test in Patients with Heart Failure with a Preserved Ejection Fraction
Source: J Clin Med. 2022 Dec 28;12(1):241. doi: 10.3390/jcm12010241 (PMC9821146; doi:10.3390/jcm12010241)

Supplementary Figure S1. Interface for processing the data extracted with the sensor for the gait kinematic analysis with the MATLAB software.

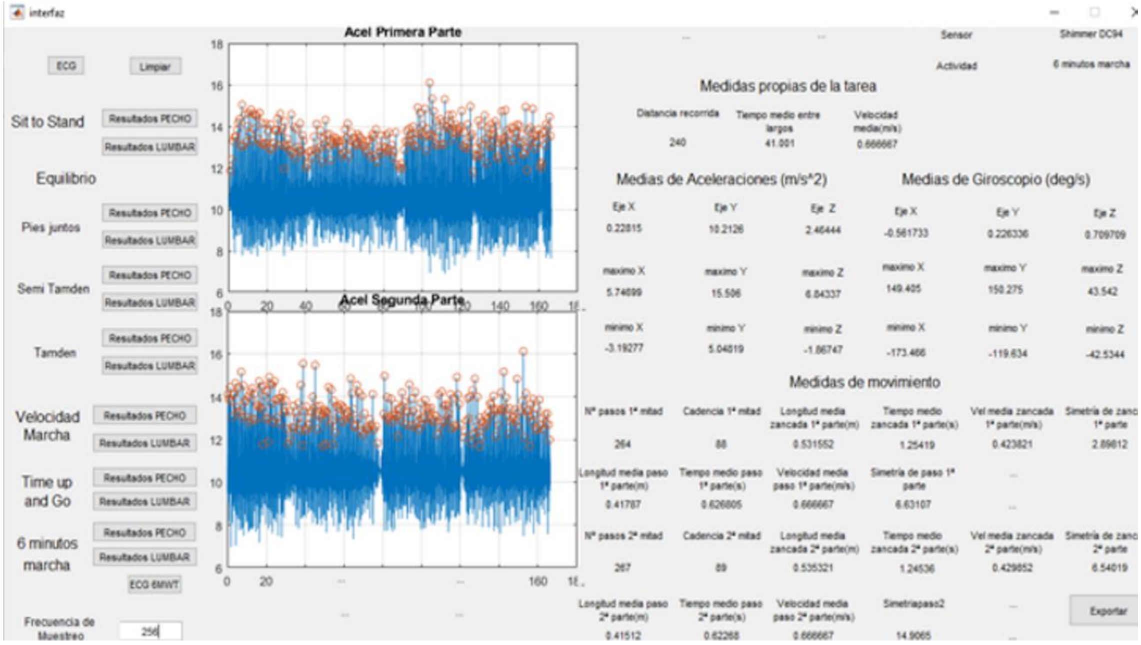

Supplement: Supplementary file 1 [file jcm-12-00241-s001.zip › jcm-2028519-supplementary/Supplementary Figure S1.pdf]
