# Supplementary material for: Kinematic Parameters That Can Discriminate in Levels of Functionality in the Six-Minute Walk Test in Patients with Heart Failure with a Preserved Ejection Fraction
Source: J Clin Med. 2022 Dec 28;12(1):241. doi: 10.3390/jcm12010241 (PMC9821146; doi:10.3390/jcm12010241)
Supplement: Supplementary file 1 [file jcm-12-00241-s001.zip › jcm-2028519-supplementary/Supplementary Table S1.pdf]

Supplementary Table S1. Participants´descriptive, anthropometric and clinical variables, as well as blood and urinary biomarkers (n=70).

	Mean (SD)	Min-Max
Age (years)	80.74 (6.11)	70.0-96.0
LVEF (%)	60.71 (4.48)	50.0-75.0
Comorbidities (n)	8.34 (1.92)	4.0-14.0
Height (m)	1.61 (0.08)	1.43-1.84
Weight (kg)	76.89 (15.11)	52.0-116.50
BMI (kg/m^2)	29.79 (6.04)	19.81-51.78
Polypharmacy (n)	10.09 (3.14)	3.0-19.0
**Blood Biomarkers**		
Hb (g/dL)	14.08 (11.55)	6.40-108.0
MCV (fL)	95.43 (19.42)	72-247
Leukocytes Count (x10^9/L)	8.10 (5.02)	2.41-45.20
Blood Platelets (x10^9/L)	217.40 (68.36)	8-415
Glucose (mg/dL)	102.09 (27.71)	61-195
Creatinine (mg/dL)	1.30 (0.60)	0.51-4.61
GF (mL/min/1,73 m^2)	51.37 (20.23)	11-90
Na^+ (mEq/L)	139.91 (3.53)	124-148
K^+ (mEq/L)	4.63 (0.50)	3.5-6.2
Cl^- (mEq/L)	101.41 (4.19)	89-111
Glycosylated hemoglobin (%)	6.32 (1.15)	4.8-10.4
Ferritin (ng/mL)	122.40 (138.89)	10-738
Transferrin Saturation (%)	21.06 (24.90)	5-214
Total Cholesterol (mg/dL)	162.31 (41.47)	98-317
LDL (mg/dL)	87.49 (29.12)	41-167
HDL (mg/dL)	46.43 (13.28)	28-90
NT-proBNP (pg/mL)	2095.99 (3354.52)	42-19118
Vitamin D (ng/mL)	23.83 (10.17)	7-50
Vitamin B12 (pg/mL)	498.02 (665.53)	103-4668
ALT (U/L)	22.60 (13.55)	8-94
CA-125 Antigen (U/mL)	43.92 (75.76)	0-525
Folic Acid (ng/mL)	8.06 (2.63)	3-16
Thyrotropin (µIU /mL)	2.46 (1.84)	0.1-12
Albumin (g/dL)	4.03 (4.02)	2.30-36
Bilirubin (mg/dL)	0.61 (0.33)	0.1-1.8
**Urinary Biomarkers**		
Urine Creatinine (mg/dL)	77.18 (34.60)	24.00-158.53

	n (Percentage)
**Drugs**	
ACE inhibitors	16 (22.90%)
ARB	46 (65.70%)
Beta-Blockers	53 (75.70%)
Sacubutril-Valsartan	0 (0%)
Ivabradine	3 (4.30%)
Ca-antagonists	19 (27.10%)
Loop Diuretics	60 (85.70%)
Mineralocorticoid Receptor Antagonist	21 (30.00%)
Thiazide	10 (14.30%)
Nitrates	19 (27.10%)
Acarboxyprothrombin	13 (18.60%)
Factor Xa Inhibitors	29 (41.40%)
Acetylsalicylic acid	22 (31.40%)
Hypoglycemic Agents	33 (47.10%)
Metformin	17 (24.30%)
SGT2I	9 (12.90%)
GLP1	2 (2.90%)
DPP4	16 (22.90%)
Insulin	16 (22.90%)
**Comorbidities**	
Arterial Hypertension	68 (97.10%)
DM	38 (54.30%)
Dyslipidemias	62 (88.60%)
Atrial Fibrillation	42 (60.00%)
COPD	19 (27.10%)
Stroke	10 (14.30%)
CRI	44 (62.90%)
OSA	14 (20.00%)
Cognitive Impairment	8 (11.40%)
Anemia	31 (44.30%)
PAD	4 (5.70%)
Depression	24 (34.30%)
Cancer Disease	10 (14.30%)
LVH	38 (54.30%)

LVD	65 (92.90%)
LAD	33 (47.10%)
Heart Valve Disease	45 (64.30%)
Aortic Valve Disease	22 (31.40%)
Mitral Valve Disease	32 (45.70%)
Tricuspid Valve Disease	17 (24.30%)
Pulmonary Hypertension	13 (18.60%)
**BMI**	
Underweight	0 (0.0%)
Normal Weight	15 (21.40%)
Overweight	28 (40.00%)
Obesity	27 (38.60%)
**NYHA**	
II	48 (68.60%)
III	22 (31.40%)
**Gender**	
female	41 (58.60%)
male	29 (41.40%)
**History of Smoking**	
No	46 (65.70%)
Yes	24 (34.30%)
**History of Alcohol**	
No	64 (91.40%)
Yes	6 (8.60%)
**Number of falls in the last year**	
0	31 (44.30%)
1	19 (27.10%)
2	8 (11.40%)
3	7 (10.00%)
> 3	5 (7.10%)
**Marital Status**	
Single	6 (8.60%)
Married	26 (37.10%)
Divorced	1 (1.40%)
Widower	37 (52.90%)
**Academic Degree/Studies Level**	
Not knowing how to read or write	10 (14.30%)

Literacy	33 (47.10%)
Primary studies	12 (17.10%)
Secondary studies	5 (7.10%)
Higher education	10 (14.30%)

LVEF: Left Ventricular Ejection Fraction; **BMI**: Body Mass Index; **Hb**: Hemoglobin; **MCV**: Mean Corpuscular Volume; **GF**: Glomerular Filtering; **Na^+**: Sodium; **K^+**: Potassium; **Cl^-**: Chlorine; **LDL**: Low-Density Lipoproteins Cholesterol; **HDL**: High-Density Lipoproteins Cholesterol; **NT-proBNP**: pro-Brain Natriuretic Peptide; **ALT**: Alanine Aminotransferase; **CA-125 Antigen**: Carbohydrate Antigen; **ACE inhibitors**: Angiotensin-Converting Enzyme Inhibitors; **ARB**: Angiotensin II Receptor Blocker; **SGT2I**: Sodium-Glucose Transporter 2 Inhibitors; **GLP1**: Glucagon-Like Peptide 1; **DPP4**: DiPeptidyl Peptidase-4; **DM**: Diabetes Mellitus; **COPD**: Chronic Obstructive Pulmonary Disease; **CRI**: Chronic Renal Insufficiency; **OSA**: Obstructive Sleep Apnea; **PAD**: Peripheral Arterial Disease; **LVH**: Left Ventricular Hypertrophy; **LVD**: Left Ventricular Dilatation; **LAD**: Left Atrial Dilatation; **NYHA**: New York Heart Association class; **SD**: Standard Deviation.
