# Supplementary material for: Kinematic Parameters That Can Discriminate in Levels of Functionality in the Six-Minute Walk Test in Patients with Heart Failure with a Preserved Ejection Fraction
Source: J Clin Med. 2022 Dec 28;12(1):241. doi: 10.3390/jcm12010241 (PMC9821146; doi:10.3390/jcm12010241)
Supplement: Supplementary file 1 [file jcm-12-00241-s001.zip › jcm-2028519-supplementary/Supplementary Table S2.pdf]

Supplementary Table S2: Descriptive kinematic parameters of the complete sample.

	**Mean (SD)**	**Min-Max**
**6MWT (m)**	246.21 (93.33)	105-540
**Lap time (s)**	50.64 (19.95)	20.00-102.86
**Gait speed (m/s)**	0.68 (0.26)	0.29-1.50
**Steps (n)**	537.94 (105.95)	298.00-716.00
**Step time (s)**	0.36 (0.12)	0.25-0.76
**Step length (m)**	0.24 (0.08)	0.09-0.59
**Step velocity (m/s)**	0.70 (0.25)	0.33-1.50
**Step cadence (steps/min)**	89.66 (17.66)	49.67-119.33
**Step symmetry ratio (step time max/step time min)**	23.67 (24.32)	2.56-106.72
**Strides (n)**	267.41 (63.32)	120.10-365.17
**Stride time (s)**	0.73 (0.23)	0.49-1.51
**Stride length (m)**	1.09 (0.59)	0.29-2.95
**Stride velocity (m/s)**	1.80 (1.27)	0.22-5.80
**Stride cadence (strides/min)**	89.14 (21.11)	40.03-121.72
**Stride symmetry ratio (stride time max/stride time min)**	10.97 (10.34)	1.69-47.40
	**n (Percentage)**	
**6MWT**		
<300m	48 (68.60%)	
>300m	22 (31.40%)	
**STOP during the 6MWT and Reason**		
Yes	29 (41.40%)	
No	41 (58.60%)	
Intolerant Dyspnea	18 (62.10%)	
Leg Fatigue or Leg Pain	10 (34.50%)	
SpO2 Drop	1 (3.40%)	

NYHA: New York Heart Association class; **SD**: Standard Deviation; **6MWT:** 6-Minute Walk Test; **HR**: Heart Rate; **SpO2**: Oxygen Saturation.
